# Draft Genome Sequence of *Streptomyces rapamycinicus* Strain NRRL 5491, the Producer of the Immunosuppressant Rapamycin

**DOI:** 10.1128/genomeA.00581-13

**Published:** 2013-08-08

**Authors:** Damir Baranasic, Ranko Gacesa, Antonio Starcevic, Jurica Zucko, Marko Blažič, Marinka Horvat, Krešimir Gjuračić, Štefan Fujs, Daslav Hranueli, Gregor Kosec, John Cullum, Hrvoje Petković

**Affiliations:** Acies Bio d.o.o., Ljubljana, Sloveniaa; Instituto de Biomedicina y Biotecnología de Cantabria (IBBTEC), Universidad de Cantabria, CSIC, SODERCAN, Santander, Spainb; Centre of Excellence for Integrated Approaches in Chemistry and Biology of Proteins (CIPKeBiP), Ljubljana, Sloveniac; Faculty of Food Technology and Biotechnology, University of Zagreb, Zagreb, Croatiad; Department of Genetics, University of Kaiserslautern, Kaiserslautern, Germanye

## Abstract

*Streptomyces rapamycinicus* strain NRRL 5491 produces the important drug rapamycin. It has a large genome of 12.7 Mb, of which over 3 Mb consists of 48 secondary metabolite biosynthesis clusters.

## GENOME ANNOUNCEMENT

*Streptomyces rapamycinicus* strain NRRL 5491 was isolated by Brazilian scientists from a soil sample collected on Easter Island. It was previously classified as *S. hygroscopicus* (1) and is often referred to in the literature as ATCC 29253. It is the only organism known to produce rapamycin (also known as sirolimus), which was initially isolated by Wyeth scientists as an antifungal agent (2). However, it proved to be a potent inhibitor of the mTOR (“target of rapamycin”) signaling pathway in mammalian cells, which endows it with a wide range of potential clinical applications (3). Currently, rapamycin is used clinically as an immunosuppressant after organ transplantation (4) and for the prevention of restenosis after stent insertion for the treatment of coronary heart disease (5). The semisynthetic derivatives of rapamycin, temsirolimus and everolimus, are approved for the treatment of renal cell carcinoma and other proliferative diseases (6, 7). Rapamycin analogues have shown promise for the treatment of cardiovascular, autoimmune, and neurodegenerative diseases (8, 9) and have even been suggested as anti-ageing treatments (10). A second gene cluster, similar to the rapamycin gene cluster, has also been reported in the strain, but a corresponding polyketide product has not yet been reported (11).

DNA sequencing was performed by use of an FLX genome sequencer (Roche). One run generated 337,804 single-end reads (average read length 467 bp) and a second run with a mate-pair library (average distance between pairs 7.8 kb) generated 146,096 reads (average read length 471 bp). The average depth of coverage of the sequence was 18-fold. For assembly we used the GS De Novo Assembler (v 2.8) program (Roche), which produced 537 contigs (of at least 100 bp in length) and one major scaffold of 12,700,734 bp. There were also nine small scaffolds (each of 2 to 3 kb in size), which seemed to be derived from assembly artifacts rather than extrachromosomal elements.

The major scaffold was scanned for potential protein-coding regions using GeneMark.hmm (12), which predicted 10,425 protein-coding genes. The *ClustScan* program (13) found 25 modular secondary metabolite clusters: 13 type I modular polyketide synthases (PKS), 5 nonribosomal peptide synthetases (NRPS), and 7 mixed PKS-NRPS clusters, including the sirolimus biosynthesis cluster. The antiSmash program (14) also found these modular clusters as well as 23 further secondary metabolite clusters of other types. Remarkably, the predicted secondary metabolite clusters account for over 3 Mb of the genome sequence.

### Nucleotide sequence accession number.

The draft genome sequence was deposited in the GenBank database under accession number CP006567.
